# Ischaemia-related cell damage in extracorporeal preserved tissue – new findings with a novel perfusion model

**DOI:** 10.1111/jcmm.12238

**Published:** 2014-02-18

**Authors:** Christian D Taeger, Wibke Müller-Seubert, Raymund E Horch, Konstantin Präbst, Frank Münch, Carol I Geppert, Torsten Birkholz, Adrian Dragu

**Affiliations:** a Department of Plastic and Hand Surgery, Friedrich-Alexander-University of Erlangen-NürnbergErlangen, Germany; b Department of Bioprocess Engineering, Friedrich-Alexander-University of Erlangen-NürnbergErlangen, Germany; c Department of Heart Surgery, Friedrich-Alexander-University of Erlangen-NürnbergErlangen, Germany; d Department of Pathology, Friedrich-Alexander-University of Erlangen-NürnbergErlangen, Germany; e Department of Anesthesiology, Friedrich-Alexander-University of Erlangen-NürnbergErlangen, Germany

**Keywords:** Caspase-3, HIF-1-α, ischaemia, tissue transplantation, perfusion, cell damage

## Abstract

Tissue undergoing free transfer in transplant or reconstructive surgery always is at high risk of ischaemia-related cell damage. This study aims at assessing different procedures using an extracorporeal perfusion and oxygenation system to investigate the expression of hypoxia inducible factor (HIF)-1-α as marker for hypoxia and of the pro-apoptotic protein Caspase-3 in skeletal muscle to elucidate potential improvements in tissue conservation. Twenty-four porcine rectus abdominis muscles were assigned to five different groups and examined after they had been extracorporeally preserved for 60 min. time. Group I was left untreated (control), group II was perfused with a cardioplegic solution, group III was flushed with 10 ml of a cardioplegic solution and then left untreated. Group IV and V were perfused and oxygenated with either an isotone crystalloid solution or a cardioplegic solution. Among others, immunohistochemistry (Caspase-3 and HIF-1-α) of muscle samples was performed. Furthermore, oxygen partial pressure in the perfusate at the arterial and venous branch was measured. Expression of Caspase-3 after 60 min. was reduced in all groups compared to the control group. Furthermore, all groups (except group III) expressed less HIF-1-α than the control group. Oxygenation leads to higher oxygen levels at the venous branch compared to groups without oxygenation. Using an extracorporeal perfusion and oxygenation system cell damage could be reduced as indicated by stabilized expressions of Caspase-3 and HIF-1-α for 60 min. of tissue preservation. Complete depletion of oxygen at the venous branch can be prevented by oxygenation of the perfusate with ambient air.

## Introduction

Ischaemia-related cell damage still represents one of the major issues throughout almost all medical disciplines, seen in daily clinical routine for instance as heart attack, stroke or transplant respectively reconstructive surgery. In case of transplant, reconstructive or trauma surgery organs, tissues or amputated extremities have to be conserved extracorporeally for minutes to hours depending to the situation given. Until reconnection to blood circulation all the tissues are at risk of ischaemia-related cell damage. Depending on the organ or tissue undergoing transplantation or replantation there is a wide range of tolerance regarding time of ischaemia. In case of poly-traumatized patients life-supporting measures are of paramount importance, whereas potential amputation injuries cannot be addressed. Another example are multi-digit amputations where the time necessary to re-establish blood circulation of the amputates exceeds their ischaemia tolerances. If the situation allows a replantation of amputated extremities an average failure rate of up to 20% is described in recent literature [1]. In transplantation medicine organ allocation mainly requires long distance transports which itself comes along with a prolonged ischaemia time and organ damage. Therefore, means of prolonging ischaemia tolerance of tissue or organs are necessary.

Skeletal muscle is known to be highly sensitive to ischaemia, as previous studies have shown that irreversible damage to skeletal muscle occurs approximately after 3–6 hrs of normothermic ischaemia [2,3]. Therefore, muscle tissue is a convenient model for studying ischaemia-related cell damage. In the field of reconstructive surgery free skeletal muscle transfer represents a standardized microsurgical tool in case of for example reconstruction of traumatized extremities. However, complications and even complete failure because of partial or complete necrosis of the transplanted tissue may occur in 5% of all cases [4]. Tissue damage caused by prolonged ischaemia after harvesting the flap is still one of the limiting factors [5,6]. At this time, the standard procedure in reconstructive surgery for flap preservation is cold storage, often in addition with a singular flush of the tissue with a saline solution. In the past, numerous studies have been set-up to learn more about causes of ischaemia-related tissue damage and optimizing conservation protocols [5,7–9]. Even though most of these studies revealed encouraging results, at present none of them essentially changed the standard procedure in daily clinical routine. There is still a lack of thorough knowledge about extracorporeal tissue preservation, which might lead to an improved conservation procedure that is applicable in daily clinical routine. This study was performed to elucidate the effects of different conservation protocols. Therefore, an extracorporeal porcine muscle perfusion study was set-up. As preservation solutions, an intracellular cardioplegic solution, developed in the 1970s by Bretschneider [10–12], and an isotone crystalloid solution were chosen [7,13]. To assess whether a treatment is beneficial for preservation, the use of immunohistochemistry with antibodies against Hypoxia inducible factor 1-α (as marker for hypoxia) and Caspase-3 (one of the initiators of the apoptotic cascade [14]) has been established [7,13]. In addition, a haematoxylin and eosin (HE) staining was performed. As there is no consensus whether the muscle is adequately oxygenated during extracorporeal perfusion [15], pO_2_, pCO_2_ and pH in the perfusate in the arterial and venous branch were measured using optical sensors.

## Materials and methods

### Laboratory animals

In this study, *n* = 12 male mature pigs (Erzeugergemeinschaft Franken Schwaben, Tierische Veredelung, Wertingen-Geratshofen, Germany) with an average weight of 35.3 kg (range 32–38 kg) were used. Both rectus abdominis muscles with the inferior epigastric arteries and veins as pedicle were harvested. The low level of anatomical variation enables constant operation procedures and its length allows good handling during perfusion [7,13]. Protocols for these experiments were approved by the animal care committee of the Friedrich-Alexander-University of Erlangen-Nuremberg.

### Anaesthesia and surgical technique

Intramuscular injection of ketanest, midazolam and atropine was used as pre-medication. Anaesthesia was started with intravenous injection of ketamin and midazolam. After intubation, total intravenous anaesthesia was performed with propofol and sufentanil. The monitoring during surgery included arterial blood pressure, oxygen saturation and heart rate as described before [13].

After disinfection, a skin incision was made starting at the xyphoideal processus continuing to caudal direction. The rectus sheath was opened and the muscle was cut cranially between the third and fourth intersection. The muscle was carefully exposed on its caudal pole following into the little pelvis, observing the inferior epigastric artery and vein. The pedicle was clipped and cut through deep inside the little pelvis, and the muscle was removed.

### Perfusion system/experimental groups

**Fig. 1:**
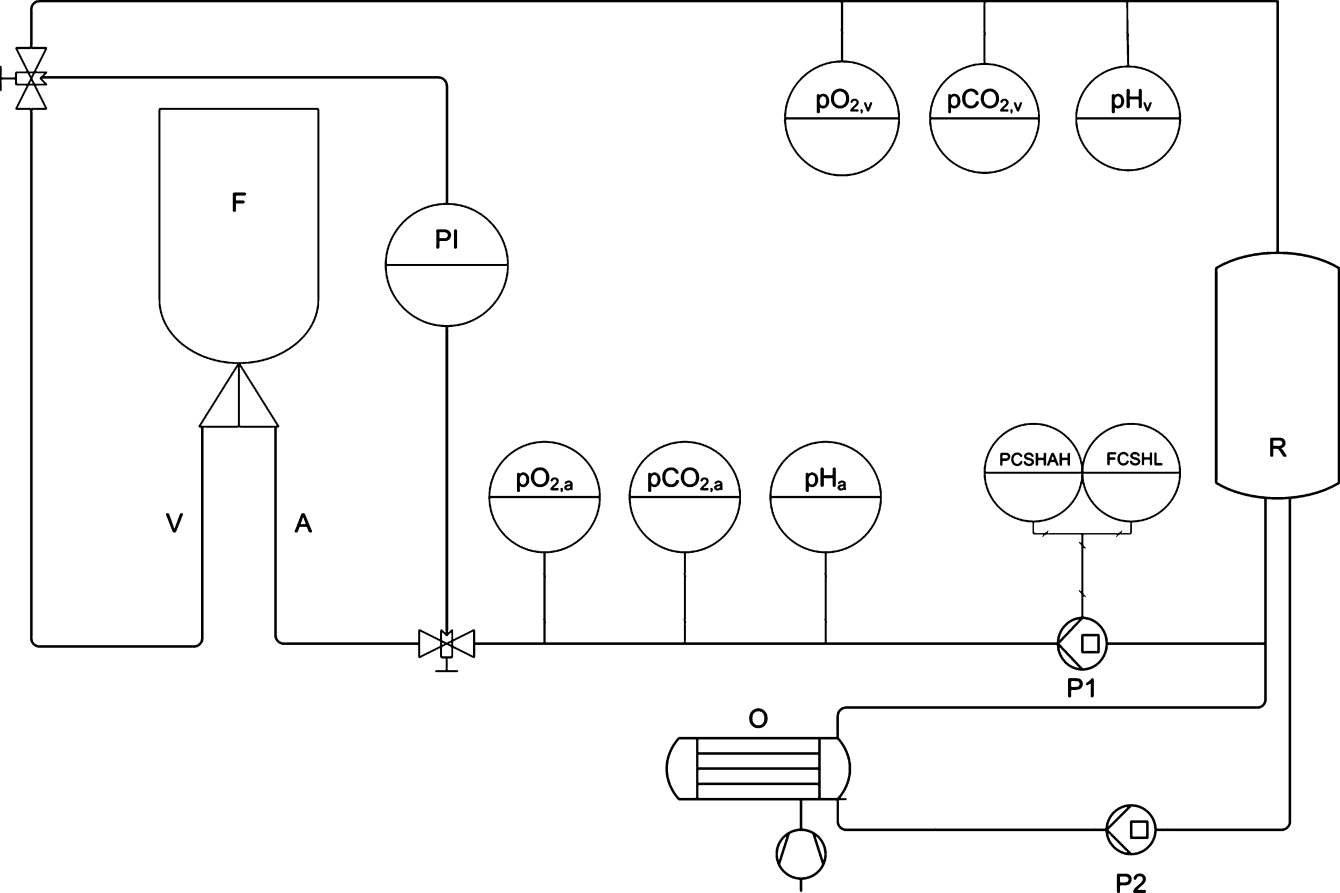
Diagram of the perfusion setting (F = muscle flap, A = arterial branch, V = venous branch, PI = pressure gauge, R = reservoir, P1/2 = pump 1/2, O = oxygenator).

*N* = 24 muscle flaps were used in this study (Table 1). After harvesting, the four flaps of group I were stored under room temperature without any further treatment (control group). The artery and vein of the five flaps in group II were cannulated with needles (arterial needles: 22-gauge, 0.9 mm in diameter, 25 mm long, flow rate of 36 ml/min., venous needles: 18-gauge, 1.30 mm in diameter, 45 mm long, flow rate of 100 ml/min.) and connected to the perfusion and oxygenation system. All needles were fixed with Perma-Hand Silk Suture 6-0 (Ethicon Inc., Johnson & Johnson, Norderstedt, Germany). Figure 1 shows a schematic model of the experimental set-up, which is an enhanced version of a previously described perfusion system [16]. Constant perfusion was performed with a flow rate (flow controlled switch high and low/FCSHL) and pressure (pressure controlled switch high and alarm high/PCSHAH) regulating pump (Fig. 1, P1; Infusomat® Space P; Braun Melsungen, Melsungen, Germany) and a flow of 10 ml/min. as previously tested [13]. Arterial and venous pressures were continuously monitored as described before [13]. The flaps of group II were perfused with a cardioplegic solution (Histidine-tryptophan-ketoglutarate Solution, HTK, Custodiol® Dr. Franz Köhler Chemie GmbH, Bensheim, Germany) for 60 min. directly after harvesting. In group III, five muscles received an arterial needle as described and were treated with a singular flush of 10 ml of the cardioplegic solution directly after harvesting and then left untreated. In group IV, five flaps were cannulated both arterially and venously to allow connection to our perfusion and oxygenation system. The additional oxygenation with ambient air was performed through a neonatal oxygenator (Fig. 1, O; SAFE Micro®, Polystan, Denmark) in a secondary circuit. Its microporous polypropylene membrane has an exchange surface of 0.33 m^2^. The primary circuit drew freshly oxygenated perfusate from the secondary circuit, which had an own pump (Fig. 1, P2). The flaps in group IV were perfused and oxygenated with a heparinized crystalloid fluid with an osmolarity of 291 mOsm/litre (Jonosteril®; Fresenius Kabi, Bad Homburg, Germany; 500 IU heparin/100 ml fluid, Ratiopharm, Ulm, Germany). In group V, five flaps were perfused and oxygenated simular to group IV with heparinized cardioplegic solution.

**Table 1:** Overview of the groups

Group	Number of flaps (*n*)	Treatment
I	4	No treatment = control group
II	5	Perfusion with HTK
III	5	Singular flush with 10 ml HTK
IV	5	Perfusion and oxygenation with Jonosteril®
V	5	Perfusion and oxygenation with HTK

### Histology

To determine the influence of the different treatments on the expression of Caspase-3 and hypoxia inducible factor (HIF)-1-α, a biopsy was taken 0 (baseline), 15, 30 and 60 min. after explantation. All samples were extracted in a standardized distance from the lower part of the muscle to achieve a high level of comparability. The biopsies were stored in formalin at ambient temperature before embedding them in paraffin. One slide per staining method was taken from the prepared sample block.

### Haematoxylin and eosin staining

Haematoxylin and eosin staining was performed according to the protocol of the Leica Autostainer XL (Leica Biosystems Nussloch GmbH, Nussloch, Germany).

### Immunohistochemistry

The slides (5–6 μm) were pre-treated with a Tris-buffer (pH 6.0) to unmask the antigen. Furthermore, antibodies against either Caspase-3 (ab4051; Abcam, Cambridge, UK) or HIF-1-α (ab16066; Abcam) were added. Before incubating at 20°C overnight, the antibodies were diluted (Caspase-3 – antibodies: 1:300, HIF-1-α- antibodies 1:1000). An alcalic phosphatase polymer kit was used as detection system (Sigma-Aldrich, St. Louis, MO, USA).

After immunohistochemistry, the slides were digitized using a digital slide scan (Mirax Midi; Carl Zeiss AG, Oberkochen, Germany). Each slide was assessed by two different interpreters to achieve double-blinding. Therefore, 10 fields (200-fold magnification, 600 × 400 μm) were distributed randomly over each slide and the total number of cells was counted by means of a histology software (Mirax Viewer, INK version 1.2). As a second step, the number of positive cells was determined in a 400-fold magnification and the percentage of positive cells in each slide was calculated. We predefined ‘positive cells’ as pink–red coloured cells and ‘negative cells’ as blue coloured. In this way, the percentage of positive cells for each slide and each group at the four different points in time was calculated.

### Measurement of O_2_, CO_2_, pH

During active perfusion, pO_2_, pCO_2_ and pH of the perfusate were continuously determined on arterial (Fig. 1, pO_2,a_, pCO_2,a_, pH_a_) and venous side (Fig. 1, pO_2,v_, pCO_2,v_, pH_v_) of the perfusion system. All parameters were determined using optical sensor systems that allow non-invasive data acquisition with fast response times (O_2_: OXY – 4 – mini, PreSens GmbH, CO_2_: pCO_2_ mini, PreSens GmbH, pH: pH-1 mini, PreSens GmbH, Regensburg, Germany). Data acquisition was performed with a rate of 4/min. The sensors contain luminescence dyes that are excited by light emitted from the transmitters. Measurement of pO_2_ is based on the principle of dynamic quenching with oxygen as the quencher. Comparing the luminescence decay time of the sensors luminophore with and without oxygen, the pO_2_ can be determined. Both the pCO_2_ and the pH Sensor use a pH sensitive luminescence dye immobilized in a buffer that uses luminescence lifetimes for calculating changes in pH. For determining CO_2_ a gas permeable membrane is included in the sensor. All Sensors are implemented in the perfusion system using flow through cells that allow parameter determination without influences of flow rate and salt concentrations in the perfusates.

### Statistical analysis

The statistical analysis was performed with IBM SPSS Statistic 20 software, IBM Deutschland GmbH, Ehningen, Germany. The different groups at time-points 15, 30 and 60 min. after harvesting were compared with the baseline, *i.e*. the average of all flaps at time 0. Furthermore, we compared a group previously treated as daily clinical routine, single flush of heparinized crystalloid solution, with the groups after 60 min. of treatment and the different groups among themselves at time-point 60 min. To determine statistical significance, the Mann–Whitney U-test for independent samples was used.

## Results

### Macroscopic findings

After 60 min. of perfusion a significant oedematous swelling of the muscle flaps was found in groups II, IV and V. Besides that no significant macroscopic changes were observed.

### Haematoxylin and eosin staining

**Fig. 2:**
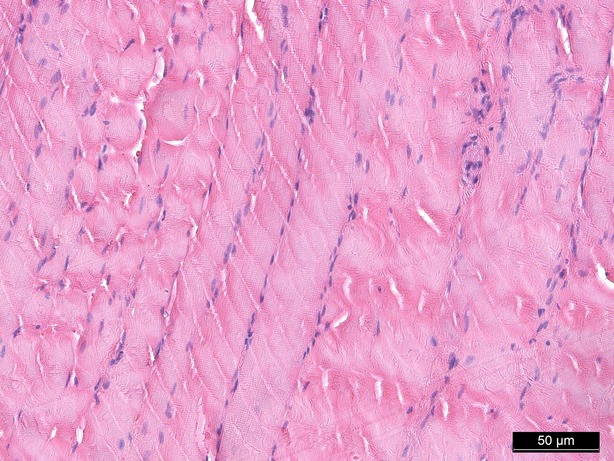
Haematoxylin/eosin staining baseline, 200-fold magnification.

**Fig. 3:**
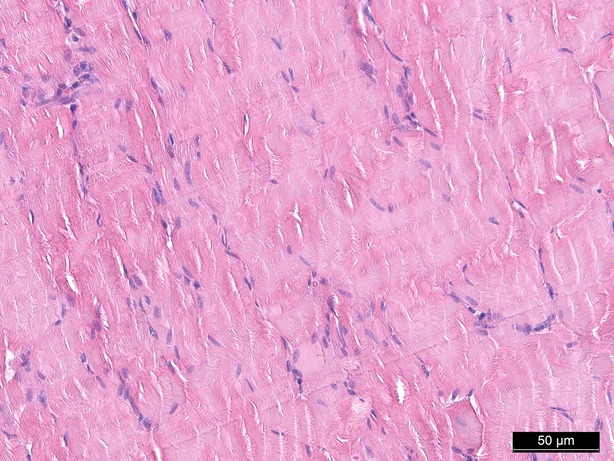
Haematoxylin/eosin staining 60 min. (control group), 200-fold magnification.

**Fig. 4:**
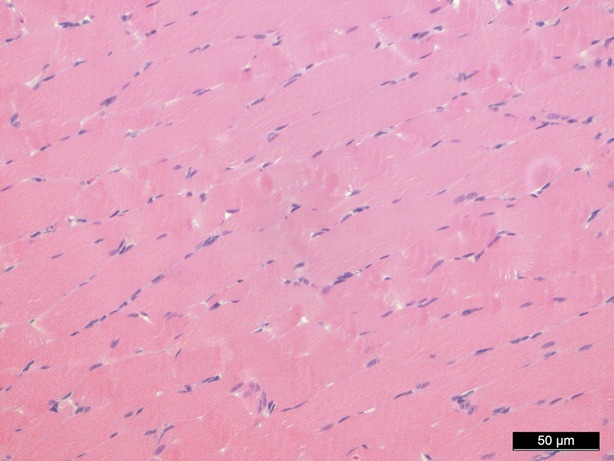
Haematoxylin/eosin staining 60 min. perfusion/oxygenation crystalloid solution, 200-fold magnification.

Haematoxylin and eosin staining was performed as a means of control and did not show significant morphological changes except for an interstitial and intracellular oedema after 1 hr of perfusion in groups II, IV and V (Figs 2–4).

### Caspase-3

**Fig. 5:**
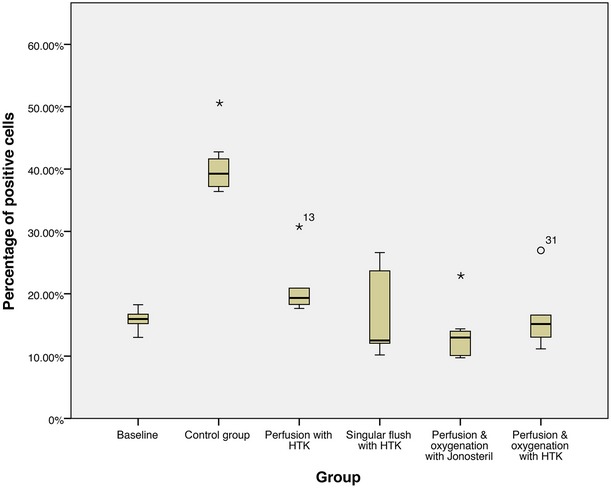
Caspase-3 immunohistochemistry, percentage of positive cells after 60 min. of treatment, * statistically significant difference to the baseline.

As shown in Figure 5, singular flush with the cardioplegic solution (group III) prevents a statistically significant increase in the expression of Caspase-3 during the different time-points 15, 30 and 60 min. compared to the baseline. Similar results have been found in the group that was treated with perfusion and oxygenation with the cardioplegic solution (group V).

Flaps of group I, which remained untreated, expressed a statistically significant higher percentage of positive cells at the three points in time 15 (*P* = 0.042), 30 (*P* = 0.006) and 60 min. (*P* = 0.006) after harvesting compared to the baseline. Similarly, perfusion with the cardioplegic solution (without oxygenation, group II) could not prevent an increase in Caspase-3 expression: at the points in time 30 (*P* = 0.030) and 60 min. (*P* = 0.005) after starting the treatment, statistically significant higher percentages of positive cells were generated in the flaps of this group compared to the baseline. In contrast, perfusion and oxygenation with the isotone crystalloid solution (group IV) reduces the expression of Caspase-3 after 30 min. (*P* = 0.048) and 60 min. (*P* = 0.010) in a statically significant way in comparison to the baseline.

Comparison of the groups (without control group) among themselves shows that nearly all treatments are equivalent after 60 min. Only perfusion and oxygenation with the isotone crystalloid solution (group IV, *P* = 0.008) shows a statistically significant better protection against an increase in Caspase-3 expression than perfusion with the cardioplegic solution (group II).

**Fig. 6:**
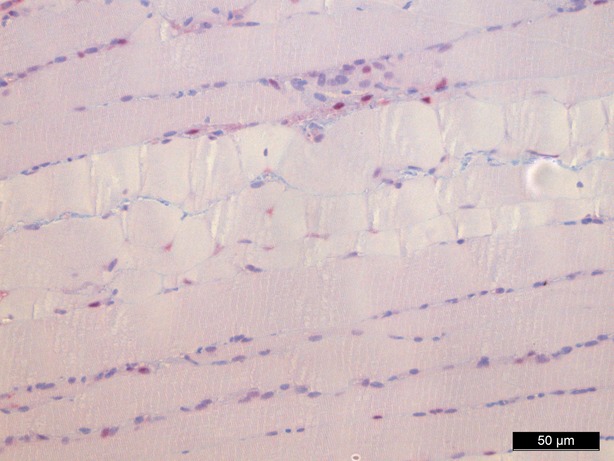
Caspase-3 staining baseline, 200-fold magnification.

**Fig. 7:**
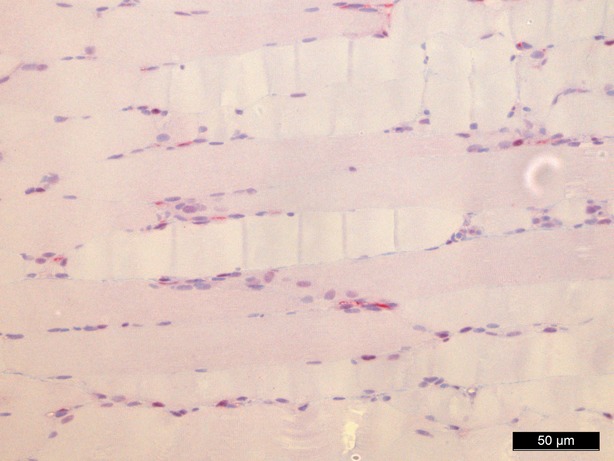
Caspase-3 staining 60 min. (control group), 200-fold magnification.

**Fig. 8:**
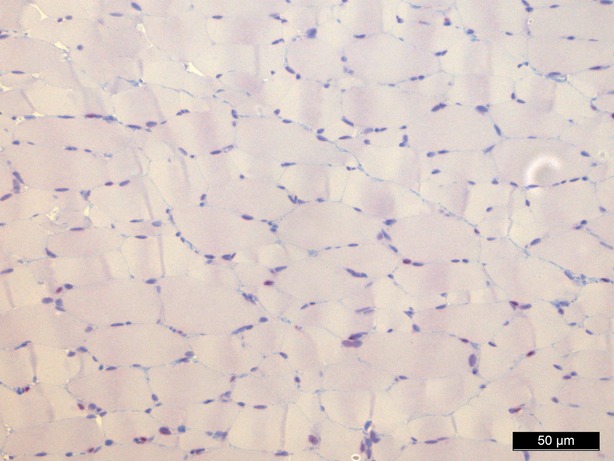
Caspase-3 staining 60 min. perfusion/oxygenation crystalloid solution, 200-fold magnification.

In conclusion, every treatment (groups II–V) reduces expression of Caspase-3 compared to the control group (group I) after 1 hr of treatment, whereby the reduction is statistically significant in all groups (*P* = 0.016 in group II–V; Figs 6–8).

### HIF-1-α

**Fig. 9:**
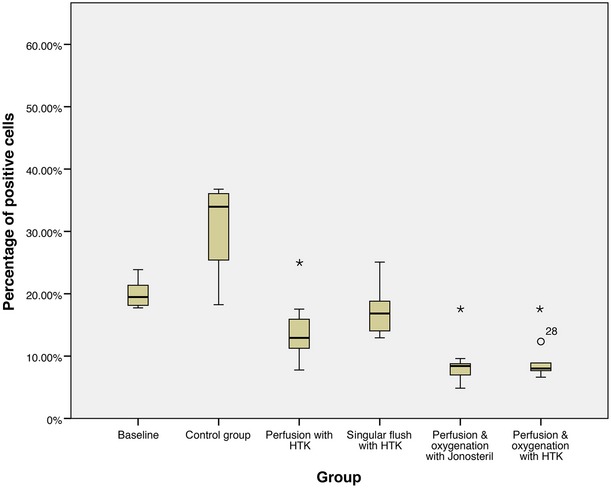
HIF-1-α immunohistochemistry, percentage of positive cells after 60 min. of treatment, * statistically significant difference to the baseline.

Group I (control group, no treatment) showed an increase in HIF-1-α expression compared to the baseline at the three points in time, however, without statistical significance (Fig. 9).

In contrast, all the other groups (groups II–V) showed statistically significant lower percentages of positive cells than the baseline. Cells of the flaps within group II (perfusion with the cardioplegic solution) expressed statistically significant less HIF-1-α after 30 min. (*P* = 0.003) and 60 min. (*P* = 0.003). The flaps of group III (singular flush with the cardioplegic solution) initially had statistically significant lower proportions of positive cells than the baseline (*P* = 0.005) after 30 min., but no statistically significant difference was found after 60 min. (*P* = 0.268). Group IV, treated with perfusion and oxygenation with an isotone crystalloid solution, showed statistically significant less cells positive for HIF-1-α at all times compared to the baseline with *P* = 0.003 (15, 30, 60 min.). Similar results were found in group V (perfusion and oxygenation with a cardioplegic solution) with statistically significant lower proportions of HIF-1-α at all three times (*P* = 0.003, 15, 30, 60 min.).

The individual groups only differ slightly from each other after 60 min. of treatment: oxygenation and perfusion with either an isotone crystalloid solution (group IV) or the cardioplegic solution (group V; both *P* = 0.008) lead to statistically lower percentages of positive cells than singular flush with the cardioplegic solution (group III). No statistically significant difference between the other groups was found.

**Fig. 10:**
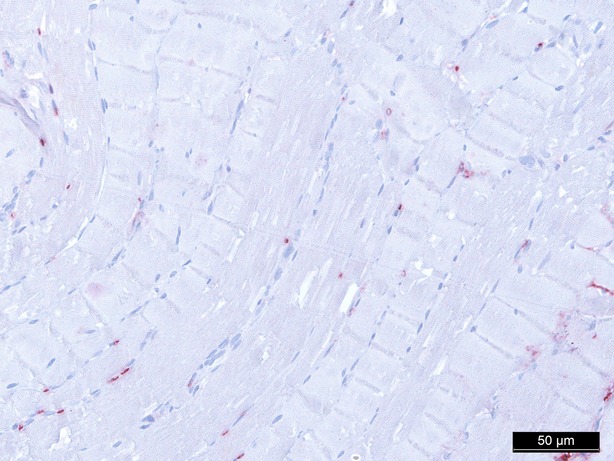
HIF-1-α staining baseline, 200-fold magnification.

**Fig. 11:**
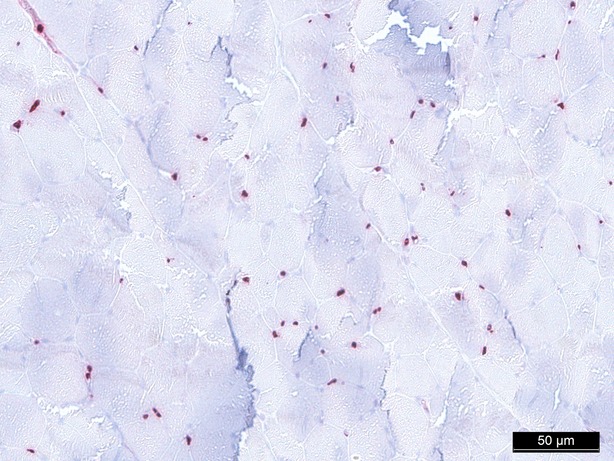
HIF-1-α staining 60 min. (control group), 200-fold magnification.

**Fig. 12:**
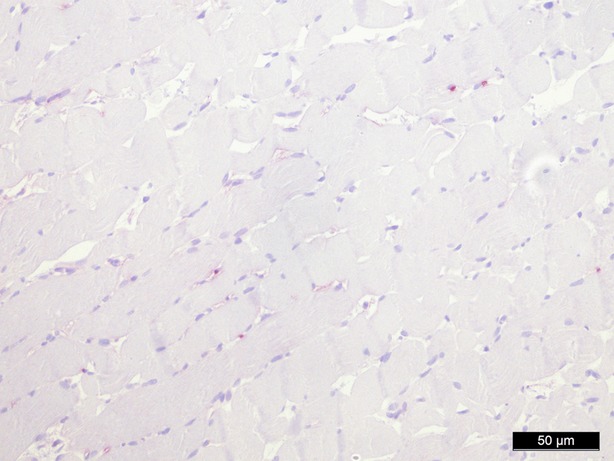
HIF-1-α staining 60 min. perfusion/oxygenation crystalloid solution, 200-fold magnification.

Comparison of the control group (group I) with the treated groups (groups II–V) confirms that all treatments (except singular flush with the cardioplegic solution, group III) decreased the HIF-1-α expression statistically significant compared to the control group (group I) after 60 min. (*P* = 0.016 in group II, IV, V; Figs 10–12).

### Summary histology

Caspase-3: Every treatment (groups II–V) showed statistically significant lower expressions of Caspase-3 after 60 min. compared to the control group (group I), whereby group IV (perfusion and oxygenation with a crystalloid solution) showed the best results.

HIF1-α: Only groups where flaps were continuously perfused either with a crystalloid or cardioplegic solution (groups II, IV, V) showed statistically significant lower levels of HIF1-α compared to the control group (group I) after 60 min.

### Oxygen partial pressure

**Fig. 13:**
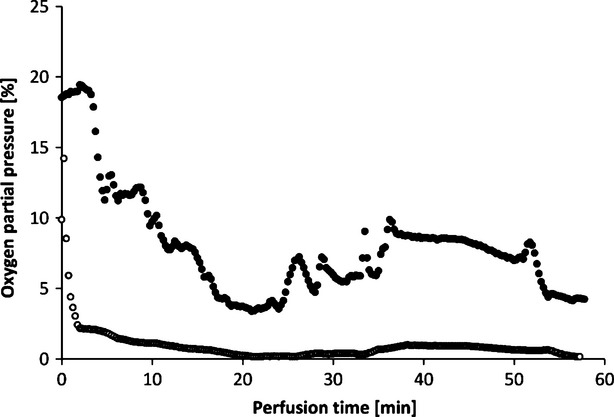
Oxygen partial pressures in arteria (•) and vein (○) in the perfusate (HTK) during active perfusion without oxygenation (example of one perfusion).

**Fig. 14:**
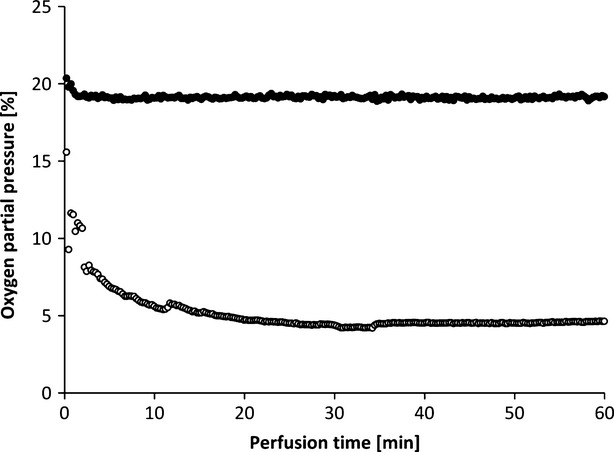
Oxygen partial pressures in the artery (•) and vein (○) and in the perfusate (HTK) during active perfusion with oxygenation (example of one perfusion).

Muscle tissue consumes high levels of oxygen even when at rest. Under stress, oxygen consumption can increase by a factor of 50 or 100 [17]. The flaps of the groups without oxygenation extracted the oxygen nearly completely during perfusion, as venous pO_2_ approximately went down to 0% pO_2_ (Fig. 13). In contrast, by supplying 21% oxygen (ambient air) *via* an oxygenator the arterial pO_2_ was stabilized and the pO_2_ at the venous branch remained at a level of 5% (Fig. 14).

**Fig. 15:**
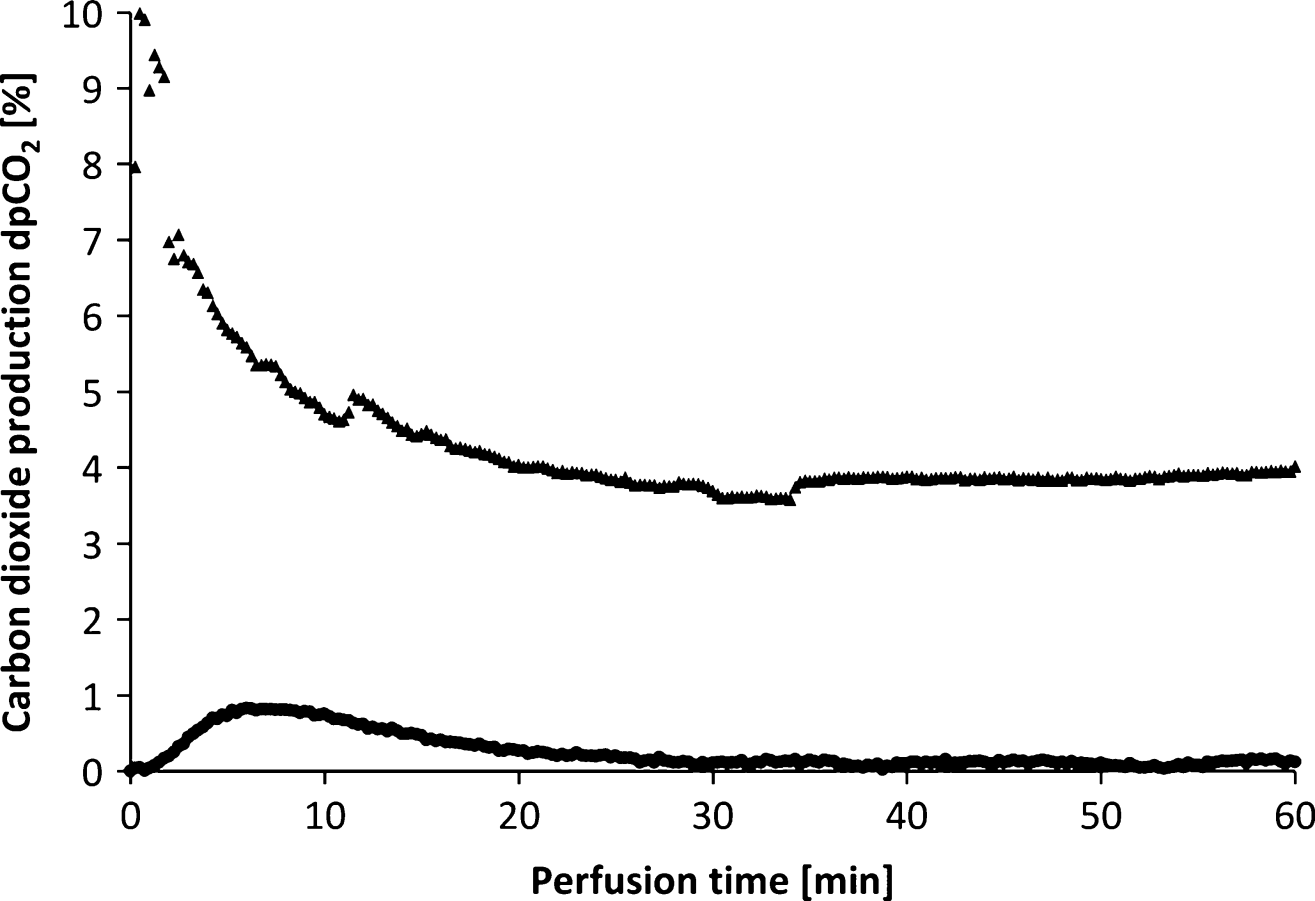
CO_2_ production of muscle tissue supplied with oxygenated (▲) and unoxygenated (•) perfusate (HTK; example of one perfusion each).

Oxygen uptake of the tissue also results in a different CO_2_ excretion into the perfusate (Fig. 15). While the maximum difference from venous to arterial pCO_2_ in the perfusate of tissues without additional oxygen supply does not reach beyond 1%, the difference in pCO_2_ with oxygenation has an initial value of 10% and still holds a medium level of 3.9% after 1 hr of perfusion.

**Fig. 16:**
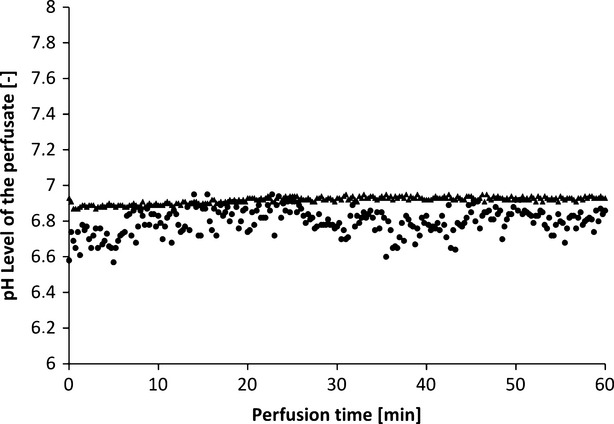
pH levels of the perfusate of muscle tissue supplied with oxygenated (▲) and unoxygenated (•) perfusate (HTK; example of one perfusion each).

Arterial and venous pH levels of the perfusate do not show any inconsistent values. Therefore, a medium value of the pH in the perfusate was used to compare perfusates in tissue with and without additional oxygen supply (Fig. 16). Both curves show a slight increase in pH over the perfusion time of 1 hr.

## Discussion

Within the last decade numerous advances were made in the field of transplant medicine. In this context, not only organ preservation, but also extracorporeal tissue conservation has made enormous progress. This was demonstrated in case of extracorporeally conserved amputated extremities as well as organs like hearts and lungs [7,18–21]. Although interesting prospects may also be seen through further advancement of tissue engineering (TE) and regenerative medicine (RM), at this time TE and RM are not clinically available and hence not yet applicable to the human patients to replace the need of human organs and tissue [22–26].

In the past, different approaches were conducted to optimize tissue conservation during ischaemia [7,12,27–29]. Currently, in reconstructive surgery flaps are mostly treated with a single flush of heparinized crystalloid solution after harvesting, followed by cold storage until their reconnection to blood circulation. This study was set-up to learn more about the influence of different parameters, which might affect the ischaemia-related cell damage of the highly ischaemia susceptible skeletal muscle and to draw conclusions for potential implications in clinical routine.

After 60 min. of continuous perfusion, a markable oedematous swelling of the muscle flaps could be found (groups II, IV, V). This finding results from the fact that the perfusates used in this study partially shift into the interstitium over the time. The addition of colloids like albumine might be rational to prevent oedematous swelling in continuous extracorporeal perfusion.

The expression of Caspase-3 – one of the most important enzymes responsible for the proteolysis of many key substrates during apoptosis [30] – was statistically significant lower in all groups after 60 min. of treatment (groups II–V) compared to the control group (group I). Furthermore, flaps treated as in clinical routine – singular flush with heparinized solution – showed a statistically significant higher level of Caspase-3 (*P* = 0.008) expression after 60 min. compared to the groups of this study [7].

As preservation solutions, a standard isotone crystalloid solution and a cardioplegic solution were evaluated. HTK, developed in the 1970s by Bretschneider (initially for use as a cardioplegia solution with a K^+^-concentration of 9 mM [10,11]), is an intracellular-type preservation solution [12]. Van der Heijden *et al*. stored skeletal muscles in different preservation solutions for 2, 8 and 16 hrs [31]. They found HTK to be superior compared to other intracellular preservation solutions such as the University of Wisconsin solution. They suggested their findings might result from the lower viscosity and the high buffering capacity of HTK [31]. Besides, Wilson *et al*. suggested that HTK might be an appropriate solution for preservation, because it significantly preserves the endothelium by its potent buffering system. Finally they mentioned that after washout of blood components, endothelial cells are the relevant cells which are in contact with the perfused solution and need to be preserved [32]. In contrast, Bastiaanse *et al*. did not find any effect on capillary perfusion after 4–6 hrs of ischaemia using pre-ischaemic perfusion of skeletal muscles with HTK [33], stasis of blood flow after reperfusion occurred in both pre-ischaemic perfused and not perfused groups [12]. Treatment with HTK in all groups in the presented study (continuous perfusion, singular flush and perfusion with oxygenation), as with all the other treatments, lead to a significant lower expression of Caspase-3 after 60 min. compared to the control group, confirming protective properties of HTK in muscle tissue.

Hypoxia inducible factor 1, a transcription factor composed of the two subunits HIF-1-α and HIF-1-β, is the key cellular survival protein during hypoxia. Hypoxia enhances the HIF-1 transactivation of target genes to help cells to adapt to changes in oxygen supply [34]. *E.g*. HIF-1 induces growth factors such as insulin-like growth factor-2, influences the angiogenesis and the glucose metabolism and has a complex role in hypoxia-induced apoptosis [35].

Hypoxia inducible factor 1-α expression changed in the different groups: all groups, except group III (singular flush with 10 ml of a cardioplegic solution), expressed statistically less HIF-1-α than the control group after 60 min. Comparison of the groups of this study with the one treated with singular flush of heparinized solution [7] – as in clinical routine – clearly demonstrates that all protocols protect against an increase in HIF-1-α (*P* = 0.008 in group III–V) expression after 60 min. of treatment in a statistically significant way.

These findings suggest that the evaluated treatments with a cardioplegic solution lead to a more adequate preservation of skeletal muscle than the standard procedure in daily clinical routine.

While the oxygenation of a cardioplegic solution did not show an advantage compared to simple perfusion, this was not the case in group IV using the isotone crystalloid solution. Perfusion with an isotone crystalloid solution and oxygenation leads to lower levels of Caspase-3 and HIF-1-α expression after 60 min. compared to the baseline, as shown in this study. Previous studies using an isotone crystalloid solution as perfusate without oxygenation, however, showed an increase in Caspase-3 and HIF-1-α levels [7]. Regarding the results of venous pO_2_ after 60 min. of treatment shows that oxygenation leads to venous pO_2_ of ∼5%, while perfusion without oxygenation decreases the pO_2_ to 0%. In conclusion, oxygenation of the perfusate with ambient air provides enough oxygen so that the muscle does not have to extract it completely. The insufficient oxygen supply may be a cause for the higher Caspase-3 and HIF-1-α values in tissue treated with an isotone crystalloid solution perfusion without oxygenation. This effect cannot be seen when perfusing with a cardioplegic solution, as described before. Here, the different composition of the perfusate seems to counteract an increase in the markers studied when offering insufficient oxygen. Its protective components for conservation, mainly Histidin as extracellular buffer, also help protecting against damage resulting from insufficient oxygen supply in the time period observed. Longer perfusion periods without oxygenation may, however, still result in an increase in Caspase-3 and HIF-1-α levels.

Our data suggest that there are methods for preventing increasing expressions of HIF-1-α and Caspase-3 when regarding conservation of free flaps. Without any further treatment after harvesting, as done with the control group, both Caspase-3 and HIF-1-α levels are markedly elevated. A singular flush with heparinized isotone crystalloid solution – as used in clinical practice – shows no effect after 60 min. [6]. A flush with a cardioplegic solution stabilizes Caspase-3 and HIF-1-α expression in free flaps compared to the baseline. This solution seems to protect the harvested tissue from detrimental effects of the ischaemia as previously discussed. This can be further monitored when using a perfusion system. While perfusion with unoxygenated crystalloid solution still results in elevated Caspase-3 and HIF-1-α levels [6], perfusing with the cardioplegic solution statistically significant decrease the HIF-1-α expression compared to the baseline and prevents an increase in Caspase-3. Furthermore, when using an oxygen regeneration circuit for providing sufficient oxygen levels, perfusion with an isotone crystalloid solution even leads to a decrease in Caspase-3 expression, as well as with using the cardioplegic solution. All groups expressed lower levels of Caspase-3 and HIF-1-α, compared to the control group after 60 min. In conclusion, conserving the harvested free flap with a singular flush of cardioplegic solution, perfusing and providing oxygen, help preserving the muscular tissue.

Although it is hard to extrapolate these experimental findings to clinical practice, the results of this study may have important implications in the future. To prevent ischaemia-related cell damage at present in clinical daily routine organs and amputated extremities are stored at a cool temperature, until reconnection to blood circulation. According to our findings, one could consider to optimize organ and tissue storage by simple means of a non-recirculating infusion: One might think about whether it would be reasonable to cannulate the arteria of an organ, an amputated extremity or free flap during ischaemia and simply perfusing the tissue by means of an infusion until anastomoses. According to our results, an ambient-air saturated solution is capable to cover the oxygen demand of a free flap. A conventional infusion is saturated with ambient air and as long as there is no recirculation an additional oxygenation is dispensable.

To prevent oedematous swelling the use of a colloid solution or the addition of for example albumin might be reasonable. One might consider setting up a study with an extracorporeal conservation time beyond 60 min. to see potential limitations of the presented technique and to learn more about the certain advantages of the different preservation protocols.
